# A Novel Approach to the Study of Pathophysiology in Patients with Obstructive Sleep Apnea Using the Iowa Oral Performance Instrument (IOPI)

**DOI:** 10.3390/jcm14134781

**Published:** 2025-07-07

**Authors:** Andrés Navarro, Gabriela Bosco, Bárbara Serrano, Peter Baptista, Carlos O’Connor-Reina, Guillermo Plaza

**Affiliations:** 1 Department of Otorhinolaryngology, Hospital Universitario de Fuenlabrada, Universidad Rey Juan Carlos, C.P. 28042 Madrid, Spain; gabrielabosco@yahoo.es (G.B.); gplaza.hflr@salud.madrid.org (G.P.); 2 Department of Otorhinolaryngology, Hospital Universitario Sanitas La Zarzuela, C.P. 28023 Madrid, Spain; 3 Department of Otorhinolaryngology, Complejo Hospitalario Universitario de Toledo, C.P. 45007 Toledo, Spain; bserranocalleja@gmail.com; 4 Department of Otorhinolaryngology, Al Zahra Hospital, Dubai 124412, United Arab Emirates; peterbaptista@gmail.com; 5 Department of Otorhinolaryngology, Hospital Quironsalud Marbella, C.P. 29603 Málaga, Spain; carlos.oconnor@quironsalud.es

**Keywords:** sleep apnea, myofunctional therapy, Iowa Oral Performance Instrument (IOPI), tongue strength, physical examination, innovation

## Abstract

**Background**: Myofunctional therapy has emerged as a treatment option for obstructive sleep apnea (OSA). The Iowa Oral Performance Instrument (IOPI) enables objective measurement of lingual and orofacial muscle strength, although it was originally designed for evaluating dysphagia. OSA is frequently associated with a hypotonic phenotype characterized by reduced strength in upper airway muscles, but its identification remains unclear. **Objective**: We evaluated the usefulness of IOPI measurements in identifying hypotonic phenotypes among patients with obstructive sleep apnea (OSA). **Methods**: We carried out a cross-sectional study analyzing the relationship between IOPI scores, sleep polygraphy metrics—such as the apnea–hypopnea index (AHI)—and findings from physical examination. In addition to the standard IOPI protocol, we introduced novel maneuvers aimed at providing a more comprehensive assessment of oropharyngeal muscle function. **Results**: Although IOPI conventional maneuvers showed no clear association with AHI or ODI, the inferior tongue maneuver showed higher awake tongue strength, with a statistically significant correlation to both AHI (r = 0.2873; *p* = 0.008) and ODI (r = 0.2495; *p* = 0.032). Performing each exercise three times yielded highly consistent results across trials (r > 0.94), but did not significantly alter the overall outcome. Interestingly, lower tongue strength values were observed in patients with a high-arched palate (*p* < 0.05), whereas no relevant associations were found with the presence of a restricted lingual frenulum or CPAP use. **Conclusions**: Incorporating specific IOPI maneuvers, especially the inferior tongue exercise, may provide additional insight into muscle function in OSA. Selective repetition is advisable for borderline values.

## 1. Introduction

Obstructive sleep apnea (OSA) is relevant because of its high prevalence and substantial impact on patients’ quality of life. It is estimated to affect between 5% and 30% of individuals, depending on the characteristics of the studied population [1]. Beyond the evident consequences on quality of life and metabolism, including daytime hypersomnolence, headache, memory impairment, increased cardiovascular risk, immune dysfunction, and worsened control of comorbid conditions such as arterial hypertension [2,3], OSA also has a significant economic impact. A systematic review of cost-related studies in sleep apnea reported highly variable costs depending on the country, ranging from an annual increase of EUR 236 in New Zealand to EUR 28,267 in the United States. Studies on European countries showed excess yearly costs ranging from EUR 1669 to EUR 5186 per patient. Furthermore, the review highlights that treatment adherence and disease control significantly influence cost, with an average increase of approximately EUR 2000 per patient per year [4].

From a pathophysiological standpoint, OSA involves a range of mechanisms that culminate in the recurrent obstruction of the upper aerodigestive tract during sleep stages, resulting in arousals and microarousals that disrupt normal sleep architecture. Various physiological factors contribute to this process, which has led to the identification of distinct phenotypes of the disease [5,6].

One critical factor in this context is the tone and strength of the oropharyngeal and oral cavity muscles, as dysfunction in these areas may increase airway collapsibility and thereby contribute to the development of apneas [7]. Accordingly, research into the relationship between muscular strength in these regions and sleep apnea has gained traction, with several studies supporting this association [8,9,10,11]. Furthermore, methods aimed at improving muscle tone, consolidated under myofunctional therapy, have shown promise in improving several clinical markers used to evaluate the severity and impact of OSA [12,13,14,15,16].

**Figure 1:**
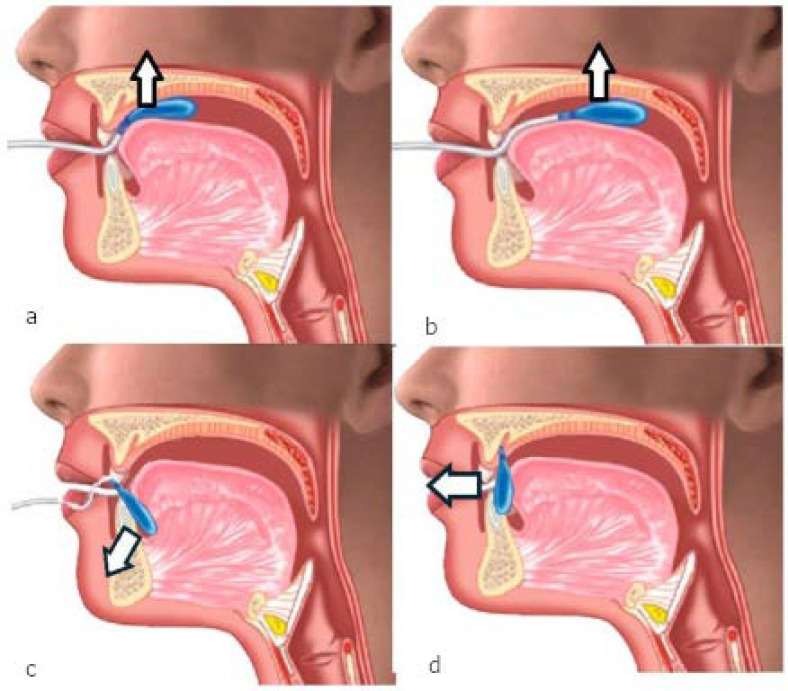
IOPI exercises to measure tongue pressure, showing direction vectors and approximate bulb positioning: (**a**) Anterior: upward pressure using the anterior tongue. (**b**) Posterior: upward pressure using the posterior tongue. (**c**) Inferior: downward pressure between the tongue’s ventral surface and the floor of the mouth. (**d**) Propulsion: forward pressure against the incisors with the tongue tip.

The Iowa Oral Performance Instrument (IOPI) is a device specifically developed to measure pressure generated by the tongue and buccinator muscles. It was initially designed for use in patients with dysphagia [17]. The original protocol consists of exercises that involve pressing the tongue tip and body against the hard palate to measure maximal pressure, and evaluating endurance by measuring how long the patients can maintain 50% of that pressure. The manufacturer recommends performing each exercise three times, with a one-minute rest between trials to minimize fatigue. Buccinator strength is assessed by placing the pressure bulb between the cheek and teeth (without biting it) and applying pressure (Figure 1) [18]. Normative reference values were established during the initial years of device use, adjusted for sex and age [19,20,21,22,23,24,25,26,27].

With growing interest in muscle tone assessment in conditions beyond dysphagia, such as dysarthria and OSA, studies have been increasingly conducted using the IOPI in these populations [12,28,29]. These studies typically employ the same exercises originally designed for dysphagia. Additionally, other tools have been developed to measure oral muscle strength, some of which correlate well with IOPI results and may offer a more economical alternative [30,31,32,33,34,35,36,37].

The aim of this study is to explore the utility of IOPI measurements in identifying hypotonic phenotypes in OSA patients.

## 2. Materials and Methods

The research protocol was developed and approved by the local ethics committee. For this study, we included patients diagnosed with OSA by the Pulmonology Department of the University Hospital of Toledo, who were referred between 2022 and 2023 to our sleep apnea clinic within the Otolaryngology Department of the same institution. Diagnoses were established through home sleep polygraphy, and the same specialist analyzed all recordings. Patients were subsequently evaluated at the sleep apnea clinic, where the same examiner carried out a structured medical history and a standardized physical examination protocol (Supplementary Materials: Examination Protocol).

During the clinical assessment, the following parameters were recorded: sex, age, neck circumference, weight, height, and smoking status (categorized as active smokers, former smokers, or never smokers). A general ENT examination was performed, including inspection of the oral cavity and oropharynx and a fiberoptic endoscopy to assess the nasal cavities, nasopharynx, posterior oropharynx, larynx, and hypopharynx. Patients were classified using the Mallampati and Friedman scales. The Müller maneuver was performed at both the retropalatal and retroglossal levels. A snoring simulation was also conducted to evaluate mouth closure response [38].

Direct measurements were obtained using a flexible ruler to determine the maximum width and height of the hard palate relative to the alveolar ridge. Additional measurements included maximum mandibular opening and the limitation observed when placing the tongue tip against the alveolar ridge behind the central incisors, following the Marchesan protocol [39,40].

Finally, lingual and buccinator muscle strength was assessed using the IOPI device. According to the manufacturer’s instructions, three maximal pressure readings were recorded in each position, allowing rest intervals between trials to minimize fatigue. Patients were asked to perform the recommended exercises: pressing the tongue tip and body against the hard palate and pressing each cheek (buccinator muscle) against the teeth without biting.

In addition to these standard exercises, two supplementary maneuvers were designed to explore other tongue muscle groups potentially more relevant to OSA pathophysiology. First, patients were instructed to place the pressure bulb behind the incisors (without biting it) and perform an anterior tongue propulsion. Afterward, they were asked to put the bulb on the floor of the mouth and press downward with the tongue (Figure 1). The standard buccinator exercises suggested by the manufacturer were also completed.

Data was analyzed using IBM SPSS Statistics Base 22.0 software (IBM Corp., Armonk, NY, USA) and appropriate statistics were employed to analyze data: Spearman’s rho and Mann–Whitney U for non-parametric variables and χ^2^ for parametric ones. Significance was considered when *p* < 0.05. Bonferroni correlations were also performed when multiple correlations were analyzed. Possible correlations between the different variables that were gathered were analyzed following physiopathological criteria. We stratified data for the high-arched palate and age and sex and still reached statistical significance.

## 3. Results

### 3.1. Population Description

**Figure 2:**
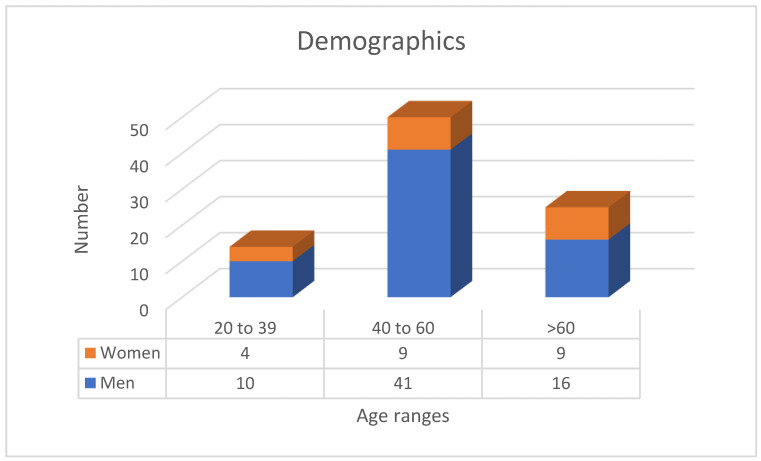
Graphic picturing the distribution of the patients in each age range differentiated by sex.

The sample consisted of 89 patients, of whom 22 were women (24.7%). The average age of the participants was 53.5 years, ranging from 21 to 76 (Figure 2). In terms of smoking history, 46% had never smoked, while 30.3% were former smokers. The mean BMI was 30.1, with a median of 28.7. Neck circumference averaged 38.1 cm, with a median value of 38 cm.

All patients underwent respiratory polygraphy, interpreted by the same pulmonologist to ensure consistency. The mean apnea–hypopnea index (AHI) was 41.5 events per hour (median: 34.2; range: 5.2 to 123). Additional respiratory parameters included minimum oxygen saturation, the oxygen desaturation index (ODI), and the cumulative time spent below 90% oxygen saturation (CT90), as detailed in Table 1.

### 3.2. IOPI Results Analysis

Several exercises were performed to assess the strength of the lingual and buccinator muscle groups using the IOPI device. The exercises described in Section 2 were used for the tongue and the buccinator muscles. One exercise per side was performed. The corresponding results are shown in Table 2.

**Figure 3:**
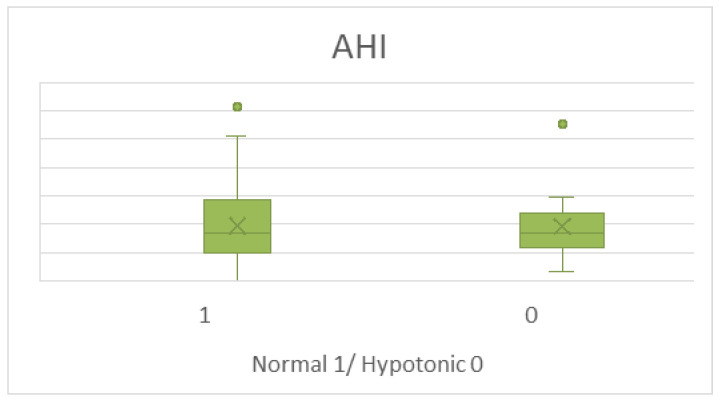
Comparison of AHI values between normotonic and hypotonic patients based on IOPI criteria using anterior maneuver.

When selecting patients with pathological values for their age group according to the manufacturer’s criteria using anterior tongue maneuver [18], 13 patients (14.6%) met this threshold and had low tongue strength, the classical hypotonic phenotype. However, in this group of hypotonic OSA patients, the mean AHI was of 38.66 (SD 26.1) events per hour and the mean ODI was 38.54 (SD 30.47), not significantly different from the remaining patients, that were 42.01 (SD 26.36) and 37.84 (SD 26.9), respectively (Figure 3).

Despite not having equivalence value standards for the remaining maneuvers, we analyzed the results in those 13 hypotonic patients and compared them to the others. The novel inferior exercise with IOPI was a mean of 31.46 kPa (SD 10.38) in the hypotonic group versus 47.31 kPa (SD 11.61) in the non-hypotonic group.

The female-to-male ratio in the normotonic group was 14/62, versus 8/5 in the hypotonic group.

As described in Section 2, each patient was asked to perform three sets of each exercise. The first value, the average, and the maximum of the three were analyzed to assess the relationship among them. The first value showed a weaker correlation with the maximum than the mean, suggesting that the lowest value is usually the first recorded. Nonetheless, no statistically significant differences were found among these values.

Table 3 presents the relationship among the anterior tongue exercises first, second, and third repetitions, including comparisons with the mean and maximum of the three repetitions.

Table 4 shows the results comparing the anterior tongue exercises first, second, and third repetitions to one another and the average and maximum values. All values showed statistical significance (*p* < 0.001).

A strong internal correlation was observed between the anterior and posterior tongue values across all these measurements (Spearman’s rho = 0.69, *p* < 0.001), as well as with those from the inferior tongue exercise (rho = 0.56, *p* < 0.001), lip exercise (rho = 0.54, *p* < 0.001), and tongue propulsion (rho = 0.6244, *p* < 0.001).

Our sample showed that elder patients had less tongue strength—a significant relationship between age and muscle strength in some of the exercises: anterior (*p* = 0.0269), posterior (*p* = 0.0104), inferior (*p* = 0.2182), propulsion (*p* = 0.0243), and buccinator right side (*p* = 0.0182). The mean value for IOPI anterior maneuver in patients < 60 years was 56.29 kPa and, for elderly patients, 47.25 kPa. Similarly, women showed lower strength—a statistically significant difference by sex was also observed, particularly using maximum values: anterior (χ^2^ = 8.257, *p* = 0.004), posterior (*p* = 0.01), propulsion (*p* = 0.0102), and buccinator left side (*p* = 0.0093), while other comparisons did not reach significance (e.g., inferior: *p* = 0.0823). These findings are consistent with prior literature highlighting sex- and age-related variability in lingual strength measures [20,26].

Lingual muscle strength did not show significant associations with CPAP use across any of the exercises analyzed: anterior tongue (exact prob = 0.98), posterior tongue (0.52), propulsion (0.61), inferior tongue (0.66), or buccinator pressure (0.54).

Likewise, no significant relationship was observed between IOPI measurements and the presence of lingual frenulum restriction. Specifically, statistical analysis across the various IOPI maneuvers yielded non-significant *p*-values: anterior tongue (*p* = 0.6809), posterior tongue (*p* = 0.050), propulsion (*p* = 0.3047), inferior tongue (*p* = 0.2610), and buccinator exercises (*p* = 0.2644). Although the posterior tongue exercise approached significance, the association was not strong enough to be considered clinically relevant within our sample. These results suggest that, in this cohort, the presence of a restricted frenulum does not have a measurable impact on tongue strength as assessed by the IOPI.

We found a statistically significant association between the presence of a high-arched palate and lower IOPI values. The comparison yielded a z-score of 1.997 (*p* = 0.0458), and this finding was consistent across most of the individual maneuvers: anterior tongue (*p* = 0.0457), posterior tongue (*p* = 0.0018), propulsion (*p* = 0.0193), inferior tongue (*p* = 0.0064), and buccinator (*p* = 0.0025). Despite the variation in *p*-values, the consistent trend indicates that individuals with a high-arched palate tend to exhibit reduced orofacial muscle strength. Clinically, it raises the question of whether a craniofacial anomaly should be routinely taken into account when interpreting IOPI results in OSA patients.

**Figure 4:**
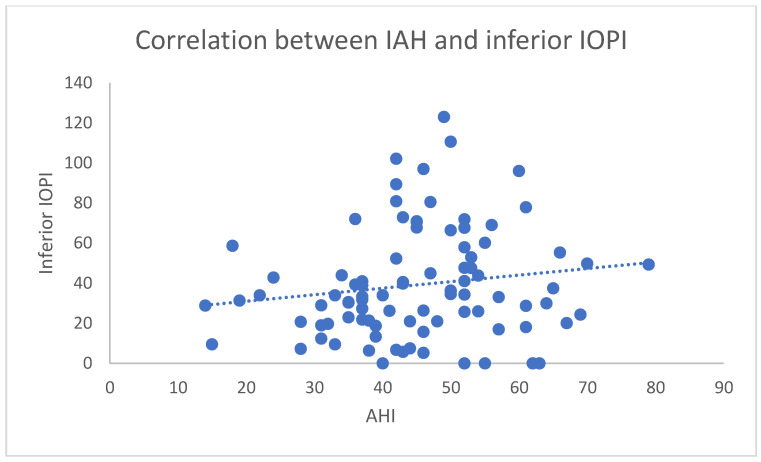
Scatter plot comparing inferior IOPI values in kPa and the AHI results.

When analyzing the relationship between IOPI maneuvers and sleep study parameters, only the inferior tongue maneuver showed a statistically significant correlation. It showed higher awake tongue strength, with a modest yet statistically significant correlation to both AHI (r = 0.2873; *p* = 0.008) and ODI (r = 0.2495; *p* = 0.032). Although the strength of these correlations was limited, the finding is relevant and no other maneuver demonstrated any significant link to objective markers of OSA severity. When IOPI is <40, average AHI was 28.6, whereas, when inferior IOPI is >40, average AHI was 48.79, a statistically significant difference (Figure 4). This may indicate that the inferior tongue maneuver activates muscle groups more directly involved in maintaining upper airway patency during sleep.

High-arched palate itself correlates significantly with AHI (z = −2.597 *p* < 0.0090) and ODI (*p* < 0.0893). As both IOPI and high-arched palate show that correlation with PG data.As both IOPI and high-arched palate show that correlation with PG data and a correlation between them, we verified our findings with stratification. When stratified by the presence of high-arched palate, the results remained significant (*p* < 0.007). A linear regression was made to determine if there were changes; inferior IOPI and high-arched palate were significant (0.013 and 0.028).

## 4. Discussion

The demographic distribution of our study population is comparable to that described in other studies on OSA [41,42]. Our results capture lingual isometric strength across a broad and diverse group of patients with varying AHI scores. A significant relationship was found in our sample between anterior tongue strength and both age and sex, which is consistent with the systematic review by Arakawa et al. [41], which includes 68 studies on IOPI, and the report of Adams et al. [17]. Both reviews also highlight that the interpretation of results may vary depending on the position of the sensor, with differences in sex and age losing significance when measuring posterior tongue strength.

In the study by Marin-Bernard et al., which aimed to establish normative reference values for the Spanish population with a mean of 49 kPa, no statistically significant differences were identified in lingual strength across age or sex groups. However, older individuals did tend to have lower values. Their inclusion and exclusion criteria mentioned respiratory conditions, but OSA was not addressed [42].

Regarding posture during the measurements, Paris-Alemany et al. reported differences in maximal tongue strength values between anterior and posterior exercises depending on head position [43]. In our study, all patients performed the exercises in a neutral head position to eliminate this potential confounder. Additionally, none of our patients engaged in intense physical activity, so the findings by Van Ravenhorst-Bell et al. [44] regarding anterior vs. posterior strength patterns in athletes should not influence our results.

Concerning exercise repetition and value selection, we found no statistically significant differences among the three repetitions in our sample. However, there was a trend for the first value to be lower than subsequent ones. Based on these results, we cannot conclude that repetition is necessary, although repeating the test may be advisable in cases of low initial values to confirm accuracy.

Currently, there is an ongoing discussion on the importance that tongue-protruding genioglossus and geniohyoid muscles and stenosing lateral pharyngeal wall muscles (stylopharyngeal and palatopharyngeal muscles) may have in each individual case [45]. Several lines of evidence suggest that using genioglossus to maintain airway patency during wakefulness is a compensatory strategy that the human applies when the standard method of maintaining airway patency has failed, and this compensatory strategy fails during sleep when the tongue relaxes [45,46,47,48].

Previous studies have reported that patients with sleep apnea tend to exhibit lower tongue strength values as measured by IOPI and similar tools, such as the tongue spoon test [31] or other instruments [49], while others have seen no significant changes with AHI using their own device [50] or the IOPI conventional anterior maneuver [51]. Electromyography (EMG) studies showed a decrease in the genioglossus function during sleep that increases upper airway resistance even in healthy people [52]. There have also been publications on EMG devices that seem to point out wake impairment of the genioglossus activity [53] and the possible interference of other pharyngeal muscles that could contribute to the upper airway dilatory mechanics regardless of the EMG of the genioglossus specifically in OSA patients [54]. In our study, values from the anterior tongue exercise were similar to those reported in other studies on OSA, around 50 kPa. The meta-analysis by Franciotti et al. [55], which includes 33 studies reporting maximal pressure values, showed that even in groups labeled as healthy, mean maximal pressures often fell below the pathological threshold. It is possible that these groups included a high proportion of undiagnosed OSA patients, which may have skewed the results, as this factor was not always accounted for or disclosed in those publications.

Our sample contains all kinds of patients suffering from OSA and, contrary to previous findings with different pressure vectors and devices [31], our results indicate that the higher the AHI values, the higher the inferior IOPI values. This conclusion aligns with recent studies stating that OSA patients may have higher IOPI values [56]. As we know, OSA is a disease that comprises different endotypes/phenotypes [5,6] so perhaps we should consider thinking that our conclusions might be contradictory depending on how relevant those hypotonic patients are in our sample. That could explain why some studies show an inverse correlation with AHI and some, like ours, show a positive correlation. In our sample we only have 13 patients that would be considered hypotonic following the validated criteria and those patients do show a non-significant negative tendency with AHI, but the vast majority of our patients were not hypotonic. Our results might have been different if we had a larger proportion of hypotonic patients. More studies should be performed selecting patients considered hypotonic to see how the correlation between AHI and IOPI behaves in that specific phenotype. Trying to explain the positive correlation in our mostly non-hypotonic patients, it may be the compensatory activation of the genioglossus that increases its wake strength, and other upper airway alteration may explain their collapses, such as dyssynchrony, stated by the Oliven group with their EMG studies [57] or Dotan et al. [58]. There are also other factors to take into account that might contribute to OSA, such as the relative positions of the hyoid and the other pharyngeal structures [59]. While the genioglossus is the main contributor to the position of the base of the tongue, there are others that contribute and play more important roles in the position of other related structures, such as the geniohyoid or digastric muscles [10,60].

Our study did not include a control group without OSA, so we are unable to determine whether our findings differ significantly from those of the general population. However, in a separate pilot study involving patients awaiting septoplasty, we identified undiagnosed cases of OSA, and some IOPI measurements in that group also showed associations with sleep apnea. Although the average values in our current cohort are somewhat lower than the manufacturer’s reference of around 60 kPa, they remain above the pathological cutoff, typically defined as the lower fifth percentile (41–43 kPa for individuals under 60 years old).

Given that our sample consists of patients with a condition that affects IOPI values, we do not believe our data are suitable for proposing general population reference ranges for the other exercises. Instead, we used absolute values to assess potential associations with OSA.

We also assessed the relationship between IOPI values and OSA severity using sleep study metrics and hypersomnolence scores. No statistically significant correlations were found between tongue strength and the AHI, ODI, or Epworth sleepiness scale, except for the inferior tongue exercise. This finding supports our hypothesis that the inferior tongue exercise may best reflect the relationship between lingual strength and OSA. Therefore, we suggest incorporating this exercise into standard protocols for assessing tongue strength in sleep apnea consultations rather than replacing existing ones.

One of the limitations of this study is its cross-sectional design, which does not allow for establishing causality, only correlations. Moreover, another limitation is the absence of a control group composed of individuals without a diagnosis of OSA. This was primarily due to the clinical setting in which the study was carried out; all participants were referred to a specialized OSA clinic after being diagnosed by the Pulmonology Department. Including healthy subjects solely for comparison was not feasible within the available resources and study design. In addition, our results are not paired with EMG to better assess the genioglossus function that may complement our data.

The goal of this study was not to define normal reference values. Instead, we aimed to understand how IOPI measurements behave in patients already diagnosed with OSA, paying particular attention to variability within the group and the possible presence of hypotonic profiles. Although the absence of a control group limits broader comparisons, the homogeneity of our sample offers a clear opportunity to examine functional differences across the spectrum of the disease.

We are carrying out follow-up work to complement our results and validate the values of the novel maneuvers. Additionally, in a larger and more diverse cohort, we are currently conducting a follow-up study aimed at validating the novel inferior and propulsion IOPI maneuvers by comparing them with electromyographic data and other objective measures of oropharyngeal muscle function.

## 5. Conclusions

Our findings support the inclusion of the inferior tongue maneuver in OSA assessment protocols, particularly in patients with borderline muscle tone. Although other IOPI parameters showed strong internal consistency, only this specific exercise demonstrated a modest but significant correlation with AHI and ODI. These results reinforce the importance of targeted assessment strategies in sleep clinics and may help refine personalized therapeutic interventions for OSA patients with orofacial hypotonia.

## Figures and Tables

**Table 1:** Oxygen saturation values from the sleep study.

Parameter	Mean	SD	Min	Max	P25	Median (P50)	P75	Units
**ODI**	37.98	27.29	1.6	124	20.0	31.25	46.1	events/hour
**Minimum SpO_2_**	78.54	8.53	51.0	92	74.0	80.0	83.5	%
**CT90**	15.6	20.51	0.0	81	1.5	5.5	20.9	% time < 90%

ODI = oxygen desaturation index; Min SpO_2_ = minimum oxygen saturation; CT90 = cumulative time below 90% saturation. Units are specified in the last column. Percentiles P25, P50 (median), and P75 reflect distribution spread.

**Table 2:** Mean results in kPa for each exercise, including average values and the maximum of the three repetitions for each task.

Exercise	First Trial (kPa)	Second Trial (kPa)	Third Trial (kPa)	Mean (kPa)	Max (kPa)
**Anterior Tongue**	46.42 (12.98)	49.80 (12.54)	49.88 (13.02)	48.70 (11.79)	53.55 (12.87)
**Posterior Tongue**	42.15 (14.41)	44.87 (13.06)	47.67 (14.24)	44.9 (12.7)	50.42 (14,24)
**Tongue Propulsion**	56.36 (14.04)	56.97 (13.05)	55.87 (14.28)	56.40 (12.81)	60.53 (13.81)
**Inferior Tongue**	37.08 (12.52)	40.01 (12.21)	40.63 (12.48)	39.24 (11)	45.00 (12.7)
**Buccinator Right**	26.26 (9.18)	28.55 (8.84)	27.85 (8.68)	27.55 (8.16)	30.97 (9.44)
**Buccinator Left**	26.52 (8.66)	26.54 (8.41)	27.28 (8.89)	26.78 (8.28)	29.22 (8.79)

All values are expressed in kilopascals (kPa). Each exercise was repeated three times. ‘Mean’ is the average of the three trials, and ‘Max’ represents the highest value obtained. Standard deviations are shown between parentheses.

**Table 3:** Correlation between repetitions and summary measures in IOPI exercises: first trial, mean, and maximum values.

Exercise	First vs. Mean	First vs. Max	Mean vs. Max
**Anterior**	0.8779	0.7642	0.9416
**Posterior**	0.9017	0.7578	0.9449
**Inferior**	0.8489	0.7716	0.9501
**Propulsion**	0.9198	0.8619	0.9670
**Right Buccinator**	0.9145	0.8302	0.9565
**Left Buccinator**	0.9222	0.8768	0.9615

**Table 4:** Correlation matrix among repetitions of the anterior tongue exercise and summary measures (mean and max) using IOPI.

	Anterior 1	Anterior 2	Anterior 3	Mean	Max
**Anterior 1**	1.0	0.7243	0.6659	0.8779	0.7642
**Anterior 2**	0.7243	1.0	0.8007	0.9196	0.9073
**Anterior 3**	0.6659	0.8007	1.0	0.9026	0.8723
**Mean**	0.8779	0.9196	0.9026	1.0	0.9416
**Max**	0.7642	0.9073	0.8723	0.9416	1.0

## Data Availability

The original contributions presented in this study are included in the article. Further inquiries can be directed to the corresponding author(s).

## Supplementary Materials

The following supporting information can be downloaded at: https://www.mdpi.com/article/10.3390/jcm14134781/s1, Examination Protocol. Video S1: IOPI: Superior Pulsion, first anterior and after posterior. Video S2: IOPI: inferior pulsion. Video S3: IOPI: Buccinator. Video S4: IOPI: Propulsion.

## Abbreviations

The following abbreviations are used in this manuscript:

ENT	Ear, nose, and throat
AHI	Apnea–hypopnea index
BMI	Body mass index
CT90	Cumulative time below 90% saturation
CPAP	Continuous positive airway pressure
IOPI	Iowa Oral Performance Instrument
ODI	Oxygen desaturation index
OSA	Obstructive sleep apnea

## References

[1] Suri T.M., Ghosh T., Mittal S., Hadda V., Madan K., Mohan A. (2023). Systematic review and meta-analysis of the prevalence of obstructive sleep apnea in Indian adults. Sleep Med. Rev..

[2] Jennum P., Ibsen R., Ibsen M., Andersen S., Kjellberg J. (2025). Long-term welfare consequences of sleep apnea in 20-64-year-olds—Influence of gender: A nationwide cohort study. Sleep.

[3] Al-Farsi H.Y., Al-Fahdi E.Y., Al-Balushi M.A., Al-Jahwari A.N., Al-Maskari A.M., Das S. (2025). Sleep Disturbances Associated with Different Systems of the Body: Underlying Mechanisms Involved and Consequences. Curr. Med. Chem..

[4] Alakörkkö I., Törmälehto S., Leppänen T., McNicholas W.T., Arnardottir E.S., Sund R. (2023). The economic cost of obstructive sleep apnea: A systematic review. Sleep Med. Rev..

[5] Raphelson J.R., Fuentes A.L., Holloway B., Malhotra A. (2024). Obstructive Sleep Apnea Endophenotypes. Sleep Sci..

[6] Eckert D.J., White D.P., Jordan A.S., Malhotra A., Wellman A. (2013). Defining phenotypic causes of obstructive sleep apnea. Identification of novel therapeutic targets. Am. J. Respir. Crit. Care Med..

[7] White D.P. (2005). Pathogenesis of obstructive and central sleep apnea. Am. J. Respir. Crit. Care Med..

[8] O’Connor-Reina C., Plaza G., Garcia-Iriarte M.T., Ignacio-Garcia J.M., Baptista P., Casado-Morente J.C., De Vicente E. (2020). Tongue peak pressure: A tool to aid in the identification of obstruction sites in patients with obstructive sleep apnea/hypopnea syndrome. Sleep Breath..

[9] Attali V., Weber M., Rivals I., Similowski T., Arnulf I., Gatignol P. (2023). Moderate-to-severe obstructive sleep apnea syndrome is associated with altered tongue motion during wakefulness. Eur. Arch. Otorhinolaryngol..

[10] Cori J.M., O’Donoghue F.J., Jordan A.S. (2018). Sleeping tongue: Current perspectives of genioglossus control in healthy individuals and patients with obstructive sleep apnea. Nat. Sci. Sleep.

[11] Oliven R., Cohen G., Somri M., Schwartz A.R., Oliven A. (2020). Relationship between the activity of the genioglossus, other peri-pharyngeal muscles and flow mechanics during wakefulness and sleep in patients with OSA and healthy subjects. Respir. Physiol. Neurobiol..

[12] O’Connor-Reina C., Ignacio Garcia J.M., Rodriguez Ruiz E., Morillo Dominguez M.D.C., Ignacio Barrios V., Baptista Jardin P., Casado Morente J.C., Garcia Iriarte M.T., Plaza G. (2020). Myofunctional Therapy App for Severe Apnea-Hypopnea Sleep Obstructive Syndrome: Pilot Randomized Controlled Trial. JMIR mHealth uHealth.

[13] Poncin W., Correvon N., Tam J., Borel J.C., Berger M., Liistro G., Mwenge B., Heinzer R., Contal O. (2022). The effect of tongue elevation muscle training in patients with obstructive sleep apnea: A randomised controlled trial. J. Oral. Rehabil..

[14] Villa M.P., Evangelisti M., Martella S., Barreto M., Del Pozzo M. (2017). Can myofunctional therapy increase tongue tone and reduce symptoms in children with sleep-disordered breathing?. Sleep Breath..

[15] Saba E.S., Kim H., Huynh P., Jiang N. (2024). Orofacial Myofunctional Therapy for Obstructive Sleep Apnea: A Systematic Review and Meta-Analysis. Laryngoscope.

[16] Carrasco-Llatas M., O’Connor-Reina C., Calvo-Henríquez C. (2021). The Role of Myofunctional Therapy in Treating Sleep-Disordered Breathing: A State-of-the-Art Review. Int. J. Environ. Res. Public Health.

[17] Adams V., Mathisen B., Baines S., Lazarus C., Callister R. (2013). A systematic review and meta-analysis of measurements of tongue and hand strength and endurance using the Iowa Oral Performance Instrument (IOPI). Dysphagia.

[18] IOPI Medical LLC (2008). Iowa Oral Performance Instrument: Users Manual. http://www.iopimedical.com.

[19] Clark H.M., O’Brien K., Calleja A., Corrie S.N. (2009). Effects of directional exercise on lingual strength. J. Speech Lang. Hear. Res..

[20] Clark H.M., Solomon N.P. (2012). Age and sex differences in orofacial strength. Dysphagia.

[21] Lazarus C., Logemann J.A., Huang C.F., Rademaker A.W. (2003). Effects of two types of tongue strengthening exercises in young normals. Folia Phoniatr. Logop..

[22] Lazarus C.L., Logemann J.A., Pauloski B.R., Rademaker A.W., Larson C.R., Mittal B.B., Pierce M. (2000). Swallowing and tongue function following treatment for oral and oropharyngeal cancer. J. Speech Lang. Hear. Res..

[23] Robbins J., Levine R., Wood J., Roecker E.B., Luschei E. (1995). Age effects on lingual pressure generation as a risk factor for dysphagia. J. Gerontol. A Biol. Sci. Med. Sci..

[24] Solomon N.P., Munson B. (2004). The effect of jaw position on measures of tongue strength and endurance. J. Speech Lang. Hear. Res..

[25] Stierwalt J.A., Youmans S.R. (2007). Tongue measures in individuals with normal and impaired swallowing. Am. J. Speech Lang. Pathol..

[26] Youmans S.R., Youmans G.L., Stierwalt J.A. (2009). Differences in tongue strength across age and gender: Is there a diminished strength reserve?. Dysphagia.

[27] Youmans S.R., Stierwalt J.A. (2006). Measures of tongue function related to normal swallowing. Dysphagia.

[28] Potter N.L., Nievergelt Y., Shriberg L.D. (2013). Motor and speech disorders in classic galactosemia. JIMD Rep..

[29] Neel A.T., Palmer P.M. (2012). Is tongue strength an important influence on rate of articulation in diadochokinetic and reading tasks?. J. Speech Lang. Hear. Res..

[30] Rodríguez-Alcalá L., Ignacio-García J.M., Serrano Angulo M.S., Casado Morente J.C., Benjumea Flores F., O’Connor-Reina C. (2022). Tongue+ protocol for the diagnosis of obstructive sleep apnoea in Quirónsalud Marbella hospital. F1000Research.

[31] Rodríguez-Alcalá L., Martín-Lagos Martínez J., O’Connor-Reina C., Plaza G. (2021). Assessment of muscular tone of the tongue using a digital measure spoon in a healthy population: A pilot study. PLoS ONE.

[32] Yoshikawa M., Yoshida M., Tsuga K., Akagawa Y., Groher M.E. (2011). Comparison of three types of tongue pressure measurement devices. Dysphagia.

[33] Yoshikawa M., Fukuoka T., Mori T., Hiraoka A., Higa C., Kuroki A., Takeda C., Maruyama M., Yoshida M., Tsuga K. (2021). Comparison of the Iowa Oral Performance Instrument and JMS tongue pressure measurement device. J. Dent. Sci..

[34] Curtis J.A., Mocchetti V., Rameau A. (2023). Concurrent Validity of the IOPI and Tongueometer Orofacial Strength Measurement Devices. Laryngoscope.

[35] Curtis J.A., Diaz C., Lee T., Rameau A. (2025). Validation of a Low-Cost Manometer to Assess of Tongue, Lip, Cheek, and Respiratory Strength: A Laboratory-Based Study. Laryngoscope.

[36] Borrmann P.F., O’Connor-Reina C., Ignacio J.M., Rodriguez Ruiz E., Rodriguez Alcala L., Dzembrovsky F., Baptista P., Garcia Iriarte M.T., Casado Alba C., Plaza G. (2021). Muscular Assessment in Patients with Severe Obstructive Sleep Apnea Syndrome: Protocol for a Case-Control Study. JMIR Res. Protoc..

[37] O’Connor-Reina C., Rodriguez-Alcala L., Ignacio J.M., Baptista P., Garcia-Iriarte M.T., Plaza G. (2023). Assessment of Muscular Weakness in Severe Sleep Apnea Patients: A Prospective Study. Otolaryngol. Head Neck Surg..

[38] Pang K.P., Kishore S., Kit J.C., Pang E.B., Chan Y.H., Keat S.J., Rotenberg B. (2016). Pang-Rotenberg sign--snoring surgery prognosticator: A prospective clinical trial of 153 patients. Laryngoscope.

[39] Marchesan I.Q. (2012). Lingual frenulum protocol. Int. J. Orofac. Myol. Myofunct. Ther..

[40] Marchesan I.Q. (2005). Lingual frenulum: Proposal of quantitative evaluation. Int. J. Orofac. Miol..

[41] Arakawa I., Igarashi K., Imamura Y., Müller F., Abou-Ayash S., Schimmel M. (2021). Variability in tongue pressure among elderly and young healthy cohorts: A systematic review and meta-analysis. J. Oral. Rehabil..

[42] Marín-Bernard E., Ruiz-López M.D., Gómez-Pozo B., Artacho R. (2024). Maximum Anterior Tongue Strength and Maximum Lip Strength in Healthy Spanish Adults: A Proposal of Reference Values. Dysphagia.

[43] Paris-Alemany A., Proy-Acosta A., Adraos-Juárez D., Suso-Martí L., La Touche R., Chamorro-Sánchez J. (2021). Influence of the Craniocervical Posture on Tongue Strength and Endurance. Dysphagia.

[44] VanRavenhorst-Bell H.A., Coufal K.L., Patterson J.A., Mefferd A.S. (2018). A comparative study: Tongue muscle performance in weightlifters and runners. Physiol. Rep..

[45] Dewald D. (2022). Rethinking the muscles of obstructive sleep apnea. Cranio.

[46] Oliven A., Tov N., Geitini L., Steinfeld U., Oliven R., Schwartz A.R., Odeh M. (2007). Effect of genioglossus contraction on pharyngeal lumen and airflow in sleep apnoea patients. Eur. Respir. J..

[47] Kubin L., Davies R., Pack A.I. (2012). Mechanisms of upper airway hypotonia. Sleep Apnea: Pathogenesis, Diagnosis and Treatment.

[48] Wang W., Di C., Mona S., Wang L., Hans M. (2018). Tongue Function: An Underrecognized Component in the Treatment of Obstructive Sleep Apnea with Mandibular Repositioning Appliance. Can. Respir. J..

[49] Kanezaki M., Ogawa T., Izumi T. (2015). Tongue Protrusion Strength in Arousal State Is Predictive of the Airway Patency in Obstructive Sleep Apnea. Tohoku J. Exp. Med..

[50] Mortimore I.L., Bennett S.P., Douglas N.J. (2000). Tongue protrusion strength and fatiguability: Relationship to apnoea/hypopnoea index and age. J. Sleep Res..

[51] Wirth M., Unterhuber D., von Meyer F., Hofauer B., Ott A., Edenharter G., Eckert D.J., Heiser C. (2020). Hypoglossal nerve stimulation therapy does not alter tongue protrusion strength and fatigability in obstructive sleep apnea. J. Clin. Sleep Med..

[52] Wiegand D.A., Latz B., Zwillich C.W., Wiegand L. (1990). Upper airway resistance and geniohyoid muscle activity in normal men during wakefulness and sleep. J. Appl. Physiol..

[53] Marghalani T.Y., Salamah R.M., Alangari H.M. (2024). A Novel Design of an Oral Appliance for Monitoring Electromyograms of the Genioglossus Muscle in Obstructive Sleep Apnea Syndrome. Life.

[54] Jugé L., Burke P.G.R., Yeung J., Knapman F., Brown E.C., Chiang A., Eckert D.J., Butler J.E., Bilston L.E. (2025). Compartmental inspiratory genioglossus electromyographic activity in supine, awake individuals with and without obstructive sleep apnoea. J. Physiol..

[55] Franciotti R., Di Maria E., D’Attilio M., Aprile G., Cosentino F.G., Perrotti V. (2022). Quantitative Measurement of Swallowing Performance Using Iowa Oral Performance Instrument: A Systematic Review and Meta-Analysis. Biomedicines.

[56] Ni A., Hao G., Chou S.Y., Chen S.C., Chang Y.J. (2025). Age-Related Changes in Tongue Strength and Endurance in Individuals with Obstructive Sleep Apnea. J. Oral. Rehabil..

[57] Oliven R., Cohen G., Dotan Y., Somri M., Schwartz A.R., Oliven A. (2018). Alteration in upper airway dilator muscle coactivation during sleep: Comparison of patients with obstructive sleep apnea and healthy subjects. J. Appl. Physiol..

[58] Dotan Y., Pillar G., Schwartz A.R., Oliven A. (2015). Asynchrony of lingual muscle recruitment during sleep in obstructive sleep apnea. J. Appl. Physiol..

[59] Li Y., Ji C., Sun W., Xiong H., Li Z., Huang X., Fan T., Xian J., Huang Y. (2023). Characteristics and Mechanism of Upper Airway Collapse Revealed by Dynamic MRI During Natural Sleep in Patients with Severe Obstructive Sleep Apnea. Nat. Sci. Sleep.

[60] Kutzner E.A., Miot C., Liu Y., Renk E., Park J.S., Inman J.C. (2017). Effect of genioglossus, geniohyoid, and digastric advancement on tongue base and hyoid position. Laryngoscope.

