# Supplementary material for: A Novel Approach to the Study of Pathophysiology in Patients with Obstructive Sleep Apnea Using the Iowa Oral Performance Instrument (IOPI)
Source: J Clin Med. 2025 Jul 7;14(13):4781. doi: 10.3390/jcm14134781 (PMC12251218; doi:10.3390/jcm14134781)
Supplement: Supplementary file 1 [file jcm-14-04781-s001.zip › 3.- Proyecto SAOSIOPI CRD v04.pdf]

CUADERNOS DE RECOGIDA DE DATOS

1. DATOS

Número asignado del paciente ....
Fecha de la exploración ....
Edad: Sexo:
~~Epworth: Stop Bang: Berlin: Quebec:~~
IOPI: Anterior: Posterior: Inferior: Labio:D I Resistencia:

Exploración Física:
Peso: Talla: IMC: PC:
Valoración esquelética facial: Normal ☐, Prognata ☐, Retrognatia ☐,
Exploración Física ORL
Frenillo lingual: longitud vertical longitud horizontal Apertura mandíbula
Marchesan apertura bucal máxima mm lengua incisivos mm relación %
Cuestionario de Hazelbaker
Paladar duro: normal ojival
Altura Ancho
Posición de lengua:
Fosas y cavum:

Orofaringe:
- Úvula: Normal ☐ Larga ☐ Amplia ☐ Larga y amplia ☐
- Amígdalas TA0 ☐ TA1 ☐ TA2 ☐ TA3 ☐ TA4 ☐
- Friedman Tongue Position (FTP) 1 ☐ 2a ☐ 2b ☐ 3 ☐ 4 ☐
- Mallampati I ☐ II ☐ III ☐ IV ☐
- Base de lengua e hipofaringe :
- Lingual Tonsil Hip. LTH 0 ☐ I ☐ II ☐ III ☐ IV
- Amígdalas linguales: normales ☐ hipertróficas ☐
- Maniobra de Müller colapso > 50%: RP ☐ RBL ☐ Epiglotis ☐

INDICE DE RONQUIDO
☐ No ronquido
☐ Respiración ruidosa no molesta para la pareja
☐ Ronquido leve/ocasional no molesto para la pareja
☐ Ronquido leve/ocasional molesto para la pareja
☐ Ronquido moderado molesto cada día para la pareja
☐ Ronquido intenso que obliga a la pareja a abandonar la habitación
☐ Ronquido muy intenso que molesta incluso a los que duermen en habitaciones contiguas

ESTUDIOS DEL SUEÑO
PR PSG
IAH ____ SaO2 Min ____ ODI ____ CT90%____ I. arousals____

Usa CPAP Si ☐ No ☐ No. Horas ______

"¿Cómo valoraría la sensación de sueño durante el día y su tendencia a dormirse involuntariamente?"

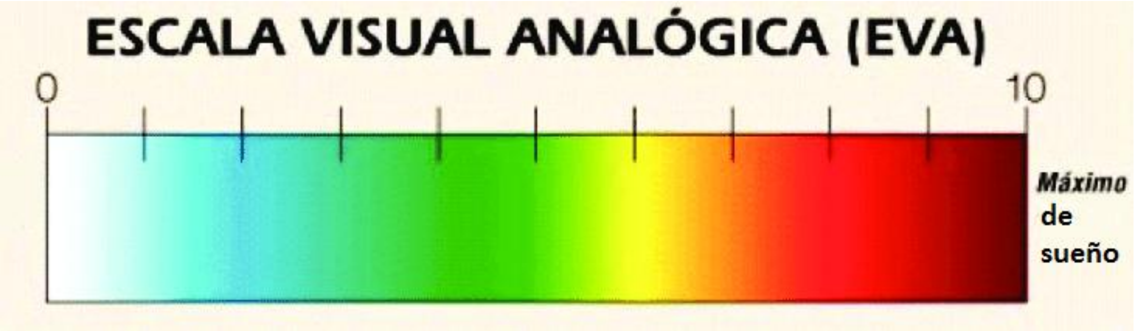

"¿Cómo valoraría la calidad de su descanso al dormir?".

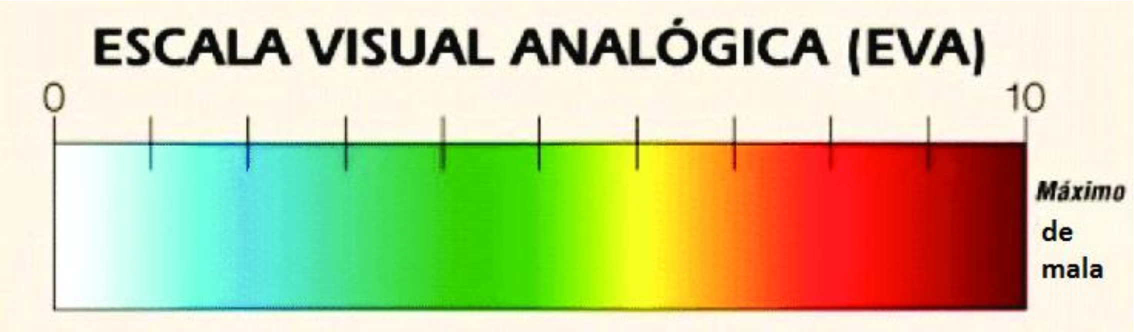

Escala Stop Bang

Escala Berlín

Escala Epworth

QUEBEC

Hiperventilación

NOSE

SNOT 22
